# Contextualizing Critical Care Medicine in the Face of Covid-19 Pandemic

**DOI:** 10.31729/jnma.5153

**Published:** 2020-06

**Authors:** Harish Chandra Neupane, Basanta Gauli, Shital Adhikari, Niki Shrestha

**Affiliations:** 1 Department of Surgery, Chitwan Medical College, Bharatpur, Nepal; 2 Critical Care Division, Chitwan Medical College, Bharatpur, Nepal; 3 Medical Education Unit, Chitwan Medical College, Bharatpur, Nepal

**Keywords:** *critical care*, *COVID-19*, *intensive care unit*, *Nepal*, *ventilator*

## Abstract

Critical Care Medicine is a specialty dealing with the comprehensive management of patients having, or at risk of developing, acute, life threatening organ dysfunction. The glaring need of critical care services and human resources for critical care have become more evident in the face of the current COVID-19 Pandemic. At this juncture, when the world is facing threat to humanity with an increasing number of deaths due to COVID 19 pandemic, the discussion about the need for ICU beds and human resources for critical care management has re-surfaced and is being increasingly realized.

In Nepal, as of 15^th^ April, 2020, there are 194 hospitals with ICU facilities. The total ICU bed strength is 1595 in 194 hospitals (which is approximately 6% of all hospital beds) and only around 50% of them are equipped with ventilators (840). These figures indicate that Nepal has approximately 2.8 ICU beds per 100,000 population. As Nepal braces to contain a major COVID-19 outbreak, the hospital capacities of the country have already come under huge pressure. If the number of confirmed cases of COVID-19 continue to rise at the current pace, the shortage of critical care facilities will become more glaring than ever before.

The current pandemic is a tremendous opportunity for health planners to accelerate action and ensure that the country is well-equipped to contain the COVID-19 pandemic. We need to be working towards infrastructure and human resource strengthening and expansion in critical care, in order to efficiently contain the pandemic.

## INTRODUCTION

Critical Care Medicine is a specialtydealing with the comprehensive management of patients having, or at risk of developing, acute, lifethreatening organ dysfunction. Critical care uses a multitude of technologies that provide support of failing organ systems, in particular, of the lungs, kidneys and the cardiovascular system. The specialty has developed expertise in the comprehensive management of disorders such as the acute respiratory distress syndrome and sepsis, however, more than the specific management of the diseases responsible for the acute illness, the common expertise of critical care is the pathophysiology and the support of organ dysfunction. Intensive care primarily aims to prevent further physiologic deterioration while the underlying disease is treated and resolves.^1^

The techniques of Critical Care Medicine now encompass not only the conventional hospital units but also emergency departments and even out-of-hospital emergency medical providers in ambulances and aircraft in which critical care monitors, measurements, and life-support devices provide care before hospital admission.^2^

The glaring need of critical care services and human resources for critical care have become more evident in the face of the current COVID-19 Pandemic that Nepal and the world is facing today.

This article will discuss the current status of critical care services andthe training opportunities for development of human resources in critical care in Nepal and will contextualize critical care medicine in the country in the face of COVID 19 pandemic.

**HISTORY OF CRITICAL CARE IN NEPA**L

In 1973, the first ICU started in Nepal at Bir Hospital, as a five bed medical ICU.^3^ This “The Old ICU” was the only ICU in the country for almost 20 years. In 1990, a six bed mixed medical surgical ICU became functional after the development of Tribhuvan University Teaching Hospital (TUTH) at Institute of Medicine (IOM), Maharajgunj. Immediately following this, TUTH added additional 5 bedded Coronary ICU and 10 additional beds of high dependency units referred to as Intermediate Cardiac Care Unit (ICCU) and Surgical ICU.^4^ Subsequently, with the increasing demands of ICU beds, critical care slowly progressed in the country.

In spite of the progress in the development of ICUSs and hospitals in Nepal over the last decade, there is paucity of definite data and publications to delineate the number of ICU beds and services in the country. Additionally, we also have a paucity of agencies to keep record of the number of ICU beds.^5^ The efficiency, services, outcomes and standards of these ICUs has rarely been published, except for very few hospitals.^6^

Determined to develop Critical Care Medicine as a specialty and improve Critical Care Services in Nepal, the Nepalese Society of Critical Care Medicine (NSCCM) was established in 2010. The NSCCM is partnering with various professional societies such as Society of Anesthesiologists of Nepal (SAN), Cardiac Society of Nepal (CSN), Society of Internal Medicine of Nepal (SIMON), and performing Continuing Medical Education (CMEs) and Workshops for upgrading themembers from various fields of medicine. NSCCM is also collaborating with Critical Care Nurses Association of Nepal (CCNAN) for enhancing Critical Care Nursing in Nepal. NSCCM is also partnering with Nepal Critical Care Development Foundation (NCCDF) for creating awareness and conducting other social activities such as “Sepsis Day” and “Hand Hygiene Campaign”.The NSCCM ICU protocols are based on latest evidence and serve asthe minimum set standard of care in ICUs of Nepal and is hoped to improve the quality of care in the ICUs of Nepal.^7^

In 2012, the Nepal Critical Care Development Foundation (NCCDF) was established. The NCCDF aims to improve the availability of Critical Care Services in Nepal and facilitates education and training which assist to improve the care for critically ill patients. NCCDF conducts various CME’s and workshops, and has been conducting‘Basic Assessment and Support in Intensive Care (BASIC) for NURSES’, ‘Infection Prevention and Control’ workshop for nurses and supports the care of patients.^8^

In 2016, Critical Care Nurses Association of Nepal (CCNAN) was officially registered as an association. From the time of its establishment, CCNAN has been conducting its activities in partnership with various other organizations such as the NCCDF and the NSCCM, aimingto enhance the skills and knowledge of critical care nurses and to promote critical care services across Nepal. CCNAN has evaluated the education and training needs of critical care nurses in the country. The executive members decided to endorse BASIC for Nurses and the Infection Prevention and Control Workshop. BASIC for Nurses specially focuses on providing an introduction to intensive care nursing for new ICU nurses. There is a strong focus on the practical needs of the new ICU nurse, with theoretical aspects being limited to those issues that are directly relevant to an understanding of clinical practice. BASIC for nurses have been conducted in many hospitals in Kathmandu valley (TU Teaching Hospital, Grande International Hospital, Nepal Cancer Hospital and Research Centre, Dhulikhel Hospital) as well as outside the valley (Biratnagar, Pokhara, Chitwan, Jhapa). CCNAN nurses are now capable to run the BASIC and the Infection Prevention and Control Workshop independently in a number of the participating hospitals. CCNAN has also started a Critical Care Nurses Instructor Training Program (CCNITP), which is a six-month training program aiming to provide long term training to the nurses interested in building a career in critical care.^9^

Up until 2012, academic training in Critical Care Medicine was a dream as there were no academic training programs in Critical Care Medicine in Nepal.^5^ As of 2014, there were around ten Intensivists who had completed their training in Critical Care either from India or abroad and were working in Nepal. This was possible because of some Universities in Canada and some institutions in India which offered international degree program for specialist physicians who wanted training in Critical Care medicine but would return back to their countries.^5^ From October 2013, a super specialty DM training program in Critical Care Medicine was started at IOM, Tribhuvan University (TU) after a formal collaboration with Royal College of Physicians and Surgeons of Canada (RCPSC) in May 2013.^5^

Another DM program in Pulmonology, Critical Care and Sleep Medicine, was started in Bir Hospital NAMS, in 2012 based on a similar program in one institute in India, PGI Chandigarh. This program was stopped in 2014 because of unavailability of preceptors at NAMS but was again introduced at BPKIHS, Dharan in 2014.^10^

A fully academic 3-year fellowship in Pulmonary & Critical Care has been initiated for certification for the first time in the history of Nepal at Chitwan Medical College. A hallmark of the full three-year academic fellowship program is the incredible diversity and refinement of the training experience, thus considerably raising the standard of patient care. The robust clinical experience and the didactic instruction equips fellows to meet the challenges of managing highly specialized case scenarios.^11^

**CURRENT STATUS OF CRITICAL CARE SERVICES IN NEPAL IN THE CONTEXT OF COVID-1**9

Nepal has faced a very slow development of critical care medicine since the establishment of the first intensive care unit (ICU) in the country in 1973 at Bir Hospital. At this juncture, when the world is facing threat to humanity with an increasing number of deaths due to COVID 19 pandemic, the discussion about the need for ICU beds and human resources for critical care management hasre -surfaced and is being increasingly realized.

The guidelines of the government endorsed in 2070 BS states that for hospitals with a bed capacity of 50 or more, of the total hospital beds, at least 5 per cent should be the ICU beds; there should be one ventilator for two ICU beds, nurse to patient ratio should be 1:1, and for the patients with severe infectious disease, there should be a provision of treatment in separate isolation bed. However, currently, not every hospital in the country is equipped with ICU facility as per the above guideline.^12^ In Nepal, as of 15^th^ April, 2020, there are 194 hospitals with ICU facilities. Nepal has 1595 ICU beds and 840 ventilators.^13^

From 24^th^ March 2020 to 30th March 2020, NSCCM conducted a telephone survey in Kathmandu Valley including all the government and private hospitals with a capacity of more than 50 beds. The survey revealed that there are roughly a total of 480 ICU beds with around 260 ventilators. Of the total ICU beds, the government-owned hospitals have only 150 beds, and the rest are in the private sector run hospitals. Majority of these ICU beds are either level I or level II and very few of the hospitals provide level III care. There are around 800 experienced or trained critical care nurses. Only a few private hospitals have ICUs with intensivist in-charge of the unit and in-house coverage round the clock; intensivist coverage in the government hospitals is a rarity. Most of the hospitals do not have proper isolation beds but a few hospitals in the private sector have private room design well enough for contact isolation.^14^

**Figure 1:**
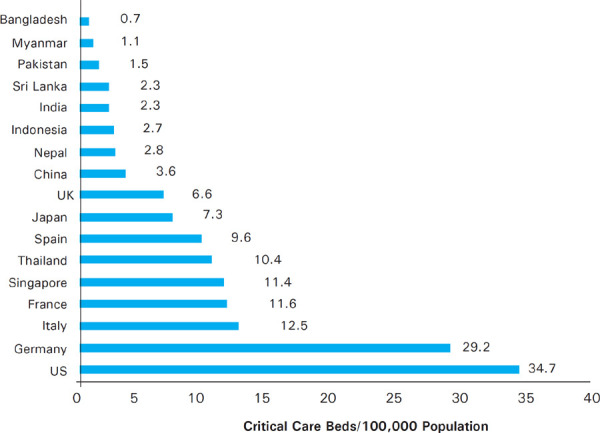
Critical Care Beds per 100,000 populations in selected countries

The infographic below pulls together data from three sources to show the number of ICU beds per 100,000 of the population in different countries. Critical care bed capacity varies widely across Asia and is significantly lower in low- and lower-middle income economies than in upper-middleincome economies and high-income economies. As per the data source, Nepal has only 2.8 ICU beds per 100,000 population (Figure 1).^15-17^

**Figure 2:**
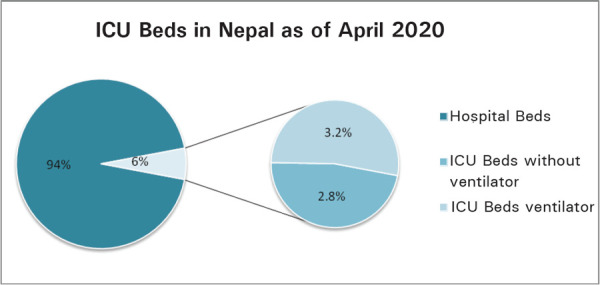
ICU Beds in Nepal.

In Nepal, at present, the total ICU bed strength is 1595 in 194 hospitals and only around 50% of them are equipped with ventilators (840). The ICU Beds comprise approximately 6% of all hospital beds. Out of this, 3.2% of the beds are ICU beds equipped with ventilators (Figure 2).^13^

It is evident from the above data that Nepal will face a critical shortage of ventilators and intensive care facilities as well as critical care human resources if the COVID-19 patients requiring critical care rise exponentially. Additionally, though no guidelines have been put forward regarding nurse patient ratio in ICUs in Nepal, most ICUs run a 1:2 to 1:3 nurse to patient ratio.^9^

As Nepal braces to contain a major COVID-19 outbreak, the hospital capacities of the country have already come under huge pressure. If the number of confirmed cases of COVID-19 continue to rise exponentially,^18^ there is a strong possibility of large percentages of the Nepalese population being admitted to the hospital and may be in need of ICU care, making the shortage of critical care facilities more glaring than ever before.

The outbreak of COVID-19 has generated concern that critically ill patients may overwhelm existing ICU bed availability. While all countries are working hard to cope with the sharp rise in infections, the existing healthcare capacity even in developed countries appears to be falling short of the real need. The government of Nepal has planned to place COVID-19 patients in public hospitals^19^ but these hospitals have few ICU beds and remain occupied most of the time with a long line of people waiting to take any vacant beds. It is unethical to remove patients from these beds in order to accommodate patient with COVID -19.

When Nepal was faced with sporadic cases in the community, rapid identification and isolation of suspected or confirmed cases to maximize containment and buy time for preparations was the key priority. However, at present, the COVID-19 cases haverisen exponentially^18^ and therefore it is already high time for strategies to be in place to manage a maximum number of COVID 19 patients by ICU expansion as well as by enhanced provision of critical care facilities as well as human resources for critical care.

**THE WAY FORWAR**D

As coronavirus disease 2019 (COVID-19) spreads across the world, the critical care fraternity must prepare for the challenges associated with this pandemic. Because of the presence of only few Intensivists and critical care nurses, the country and healthcare system demands more number of trained Intensivists and critical care nurses to manage critically ill patients across hospitals in Central, Provincial and Local Levels.Education is important for upskilling health care workers, effective infection control, and identifying technical and logistic challenges with ICU care.^20^ In addition, periodic refresher re-training is required to ensure staff readiness and proficiency. We need to identify nurses,specifically nurses working in the post-anaesthesia care unit and postoperative ward and other paramedical staff and provide them with focused short-term training to take care of critically ill patients.^14^

ICUs should be equipped to expand immediately by at least 20% above baseline capacity. During a pandemic, however, significantly higher surge ICU capacity is needed and critically ill patients may need to receive care outside of a traditional ICU. The planned response will depend on available resources, and trigger targets for activation of each phase of response should be defined early.^20^ In a workshop conducted in July 2014, the NSCCM in collaboration with Ministry of Health had planned to accredit ICUs based on their facilities as Level I, II, and III. After the workshop, the then president of the society, Prof. Moda Nath Marhatta, made recommendation to the Ministry of Health to work in collaboration with NSCCM to develop certain Level ICUs in District, Zonal Hospitals and Tertiary Centers. They also recommended for a governing body for accreditation of these ICUs.^5^ The time has come to revisit those recommendations at this critical phase when COVID-19 is rising exponentially in Nepal. Therefore, for small district hospital, private nursing home (up to 50 bedded hospital), Level I ICU needs to be set up. For large general hospital (up to 100- 150 bedded hospital), a Level II ICU needs to be set up. For tertiary level hospital, medical college (>150 bedded hospital), a Level III ICU needs to be set up.^21^

However, constructing new ICUs is a challengingtask for Nepal in the midst of COVID-19 pandemic. A feasible alternative would therefore be to upgrade the existing facilities like postoperative care beds to ICU beds. As a feasible alternative, operating rooms can be used to manage critically ill patients using anesthesia machines as ventilators if we fall short of ICU ventilators. Similarly, Post-Anesthesia Care Unit can also be utilized.^14^

Infection prevention and control is essential to protect both patients and health care workers. The vulnerability of health care workers could be seen from the experience from SARS. It was also seen that an uncontrolled in-hospital outbreak can devastate an entire hospital’s services within days. Critical care health care workers are at high risk considering the higher exposure dose from aerosol-generating procedures and longer periodsof patient contact. In addition, the reproductive numberfor SARS-CoV-2 (between 2 and 2.5) and thereforetransmissibility is significantly higher than seasonal influenza.^20^ Therefore, personal protective equipment for health care workers, which are already in short supply, need to be arranged adequately and in a timely manner. This is of special importance as we have very limited skilled human resources including intensivist and critical care nurses.^14^

In addition, since a large proportion of the healthcare capacity lies in the private sector, public-private partnership is a critical component of the response to COVID 19 in Nepal. There is a need to develop public-private partnership preparedness plans at all Central, Provincial and Local Levels to rapidly develop the surge bed capacity.

While we should hope for the best, we must be prepared for the worst. The current pandemic in Nepal is a tremendous opportunity for health planners to accelerate action and ensure that the country is well-equipped to contain the COVID-19 pandemic. We need to be working towards infrastructure and human resource strengthening and expansionin critical care, in order to efficiently contain the pandemic.

## CONCLUSIONS

Nepal has faced a very slow development of critical care medicine since the establishment of the first intensive care unit (ICU) in the country in 1973 at Bir Hospital. At this juncture, when the world is facing threat to humanity with an increasing number of deaths due to COVID 19 pandemic, the discussion about the need for ICU beds and human resources for critical care management has re -surfaced and is being increasingly realized. As Nepal braces to contain a major COVID-19 outbreak, the hospital capacities of the country have already come under huge pressure. If the number of confirmed cases of COVID-19 continue to rise exponentially, there is a strong possibility of large percentages of the Nepalese population being admitted to the hospital and in need of ICU care, making the shortage of critical care facilities more glaring than ever before. The current pandemic in Nepal is a tremendous opportunity for health planners to accelerate action and ensure that the country is well-equipped to contain the COVID-19 pandemic. We need to be working towards infrastructure and human resource strengthening and expansion in critical care, in order to efficiently contain the pandemic.

## Conflict of Interest:

**None.**
